# Design of the pilot, proof of concept REMOTE-COVID trial: remote monitoring use in suspected cases of COVID-19 (SARS-CoV-2)

**DOI:** 10.1186/s40814-021-00804-4

**Published:** 2021-03-05

**Authors:** Fahad Mujtaba Iqbal, Meera Joshi, Gary Davies, Sadia Khan, Hutan Ashrafian, Ara Darzi

**Affiliations:** 1 Division of Surgery & Cancer, St Mary’s Hospital, 10th Floor Queen Elizabeth the Queen Mother Wing (QEQM) St Mary’s Campus, London, W2 1NY UK; 2 West Middlesex University Hospital, Twickenham Road, Isleworth, TW7 6AF UK

**Keywords:** Remote-sensing technology, Protocol, Clinical trial, Patient deterioration, Monitoring, Ambulatory

## Abstract

**Background:**

The outbreak of SARS-CoV-2 (coronavirus, COVID-19), declared a pandemic by the World Health Organization (WHO), is a global health problem with ever-increasing attributed deaths. Vital sign trends are routinely used to monitor patients with changes in these parameters often preceding an adverse event. Wearable sensors can measure vital signs continuously (e.g. heart rate, respiratory rate, temperature) remotely and can be utilised to recognise early clinical deterioration.

**Methods:**

We describe the protocol for a pilot, proof-of-concept, observational study to be conducted in an engineered hotel near London airports, UK. The study is set to continue for the duration of the pandemic. Individuals arriving to London with mild symptoms suggestive of COVID-19 or returning from high-risk areas requiring quarantine, as recommended by the Public Health England, or healthcare professionals with symptoms suggestive of COVID-19 unable to isolate at home will be eligible for a wearable patch to be applied for the duration of their stay. Notifications will be generated should deterioration be detected through the sensor and displayed on a central monitoring hub viewed by nursing staff, allowing for trend deterioration to be noted. The primary objective is to determine the feasibility of remote monitoring systems in detecting clinical deterioration for quarantined individuals in a hotel.

**Discussion:**

This trial should prove the feasibility of a rapidly implemented model of healthcare delivery through remote monitoring during a global pandemic at a hotel, acting as an extension to a healthcare trust. Potential benefits would include reducing infection risk of COVID-19 to healthcare staff, with earlier recognition of clinical deterioration through ambulatory, continuous, remote monitoring using a discrete wearable sensor. We hope our results can power future, robust randomised trials.

**Trial registration:**

ClinicalTrials.gov Identifier: NCT04337489.

## Background

The recent outbreak of SARS-CoV-2 (COVID-19), declared a pandemic by the World Health Organization (WHO) and a growing global health problem, has stretched resources, creating pressures within the National Health Service (NHS) with implications for patient safety [1]. The UK Chief Medical Officers have raised the risk to the UK to high, with ever increasing confirmed cases and attributed deaths [1].

High-risk travellers with suspected or confirmed cases of COVID-19 are likely to be transferred immediately to a hospital. However, travellers with milder symptoms or returning from high-risk areas, a 2-week period of observation/quarantine may be required, in accordance with the Public Health England recommendations [2]. The rate of clinical deterioration for individuals suffering with COVID-19 remains unknown; given that widespread vaccine deployment remains imminently unforeseeable, novel strategies are required in approaching this pandemic.

Vital sign trends (heart rate, respiratory rate, blood pressure, temperature, oxygen saturations) are routinely used for monitoring hospital patients [3]. Clinical deterioration may be recognised through changes in these parameters and often precedes an adverse event [4, 5]. As such, the National Institute for Health and Care Excellence (NICE) and the Royal College of Physicians (RCP) recommend that all patients have their vital signs recorded every 12 h as a minimum [6, 7].

Across the National Health Service (NHS), the use of the National Early Warning Score 2 (NEWS), a ‘track and trigger’ warning score, has been implemented in accordance with the RCP to guide on escalation protocols and monitoring frequency of vital signs [7]. Accordingly, heart rate (HR), respiratory rate (RR), temperature, blood pressure, oxygen saturations, and level of consciousness are assessed every 4–6 h with more frequent monitoring for acutely unwell patients.

Progression in non-invasive digital technologies have renewed potential for remote-monitoring solutions [8, 9]. Wearable sensors offer an opportunity for sensor-alerting systems to continuously monitor vital parameters remotely, recognise early deterioration and support clinical decision making, allowing people to receive monitoring outside of expensive hospital facilities, in resource-limited hospitals, and utilising alternate sites in crisis scenarios [10].

Previous studies have demonstrated acceptability and practicability of continuous monitoring using wearable sensors on general surgical and medical wards in the UK and the Netherlands [11, 12]. Initial work by Downey et al. was limited by imbalanced randomisation leading to significant baseline differences across the two trial arms and failure to adjust for these in their analyses [11]. Nevertheless, the wearable sensor seemed to demonstrate feasibility in hospital settings. Qualitative analyses, through semi-structured interviews for patients and healthcare staff, similarly favoured continuously the notion of continuous vital sign monitoring in general wards [12]. It should be noted that many of the patients interviewed were admitted for malignant disease which is likely to influence qualitative outcomes. Delivery of healthcare outside of hospital facilities (e.g. in hotels) is theoretically possible through continuous remote monitoring of vital signs but has yet to be studied; given the global pandemic and fear of future waves, evaluation of its viability is justified.

Here, we describe the design of our pragmatic trial, exploring the feasibility of remote-monitoring systems in suspected cases of COVID-19 at a hotel, primarily as earlier recognition of clinical deterioration.

## Methods

### Overall design

This pragmatically designed, pilot, proof-of-concept, observational study was reviewed and approved by the London–Queen Square Research Ethics Committee (IRAS: 281757), and this protocol was developed in accordance with recommendations from the Standard Protocol Items: Recommendations for Interventional Trials (SPIRIT) guidelines [13]. The objective is to determine the feasibility of remote healthcare delivery in a hotel with rapid implementation in the COVID-19 era. Feasibility will be tested through rate of participation and number of vital alerts generated.

**Fig. 1:**
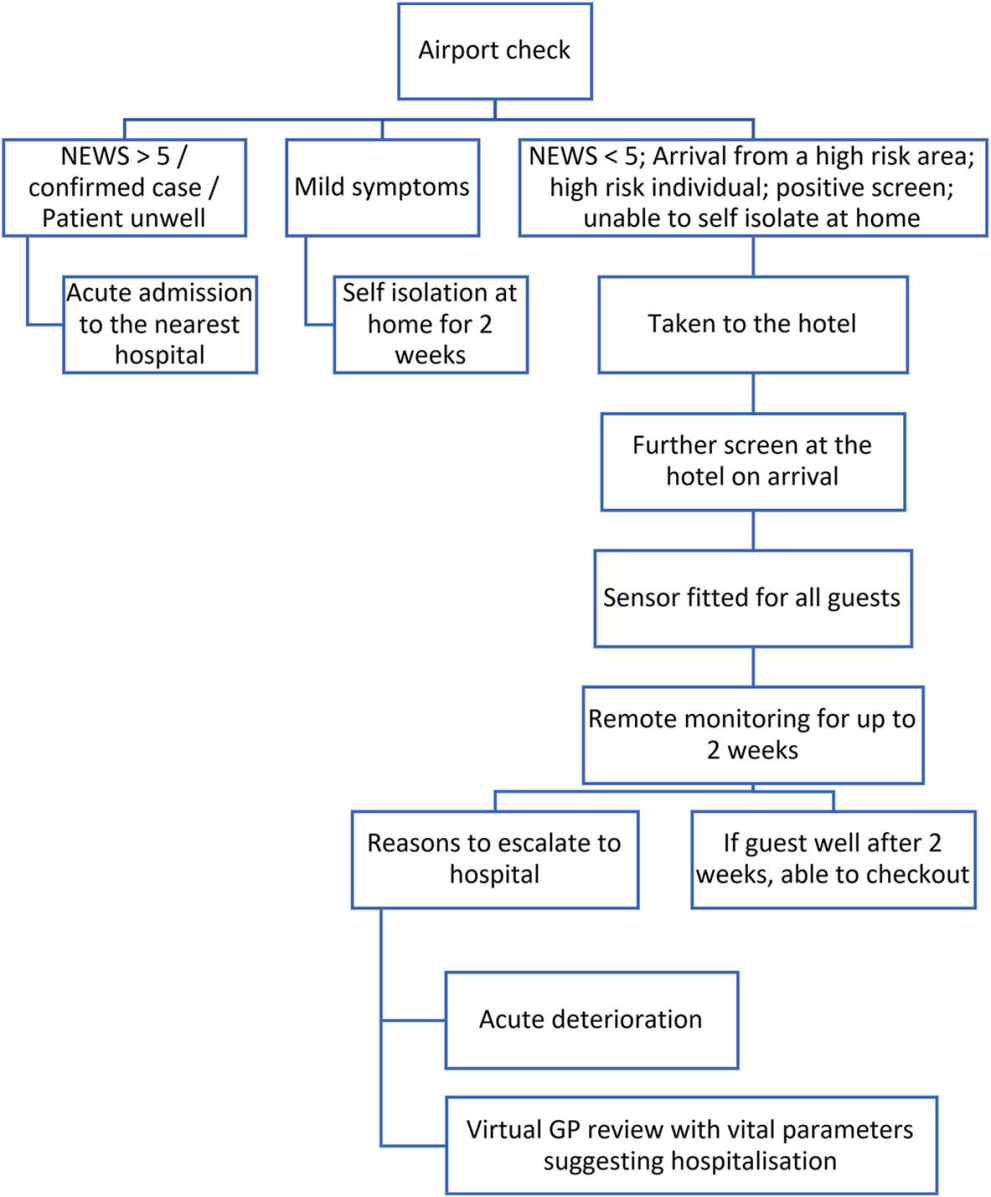
Potential escalation pathway; NEWS, National Early Warning Score; GP, general practitioner

The SensiumVitals™ (Sensium Healthcare Ltd., UK) system can be engineered in the building (hotel) for use with the wearable sensor. All participants would be fitted with the sensor on arrival and would always be worn. A designated area within the facility would act as a central hub allowing for remote monitoring to occur by healthcare staff. Alerts would be generated, in accordance with NEWS parameters (Table 1) but can be individually tailored, to identify deteriorating participants. Participants can be escalated to hospitals should rapid deterioration in vital signs be noted; ambulance services and paramedics will be present on site to facilitate this. The decision to escalate will come from a senior nurse, present on site, or a general practitioner contactable at all times. The temperature alert threshold was lowered to the lowest score in the NEWS chart, allowing for earlier detection and improved sensitivity [14]; this decision was made from previous work which demonstrated varying limits of agreement for temperature when compared to nursing observations [15]. Escalation protocols will be developed and trialled, allowing staff to address issues as they arise (Fig. 1).

**Table 1:** Criteria for generating vitals alert

Parameter	Alert threshold
Respiratory rate (breaths per minute)	≥ 25
Temperature (Celsius)	≥ 38.1
Heart rate (beats per minute)	≥ 131

### Wearable sensor system

**Fig. 2:**
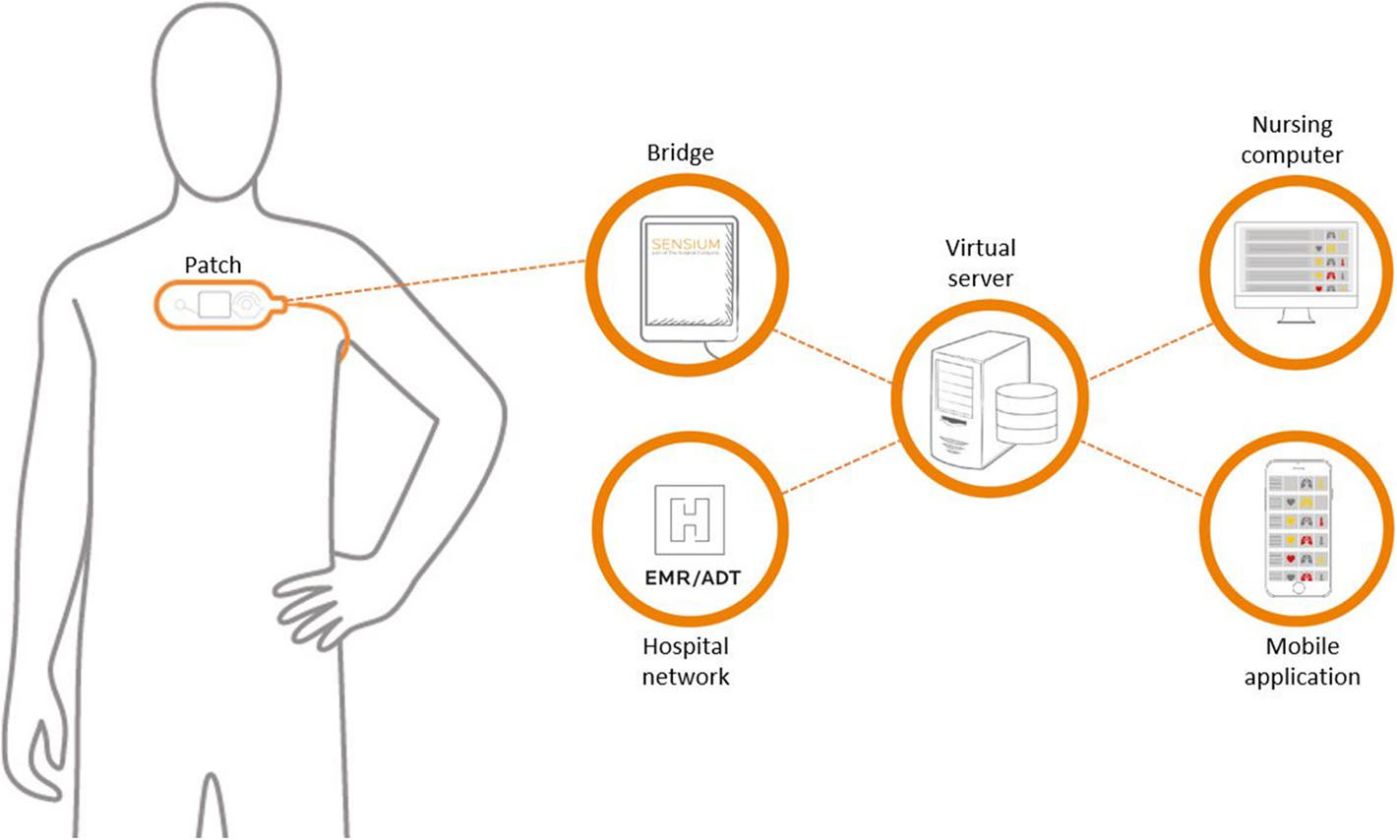
Monitoring system for SensiumVitals™ wearable sensor

SensiumVitals™ by the Surgical Company has produced a lightweight, waterproof, single-use, wearable wireless ‘patch’ with a battery life of 5 days measuring vital signs every 2 min. The sensor is attached to an individual’s chest with two ECG electrodes, recording HR and RR, and a wire is attached around the individual’s back, measuring axillary temperature. The sensor is FDA approved and CE marked. Data are transmitted through radiofrequency and dedicated intranet hotspots. Physiological parameters can be viewed by clinical staff on a mobile device/desktop computer, allowing trends towards deterioration to be noted, with tailored alerts generated for deteriorating individuals in accordance with NEWS (Fig. 2).

All sensor data collected by the SensiumVitals® system will be stored on a created secured hotel network; access to which is restricted to research personnel. Given that the hotel acts as an extension to a healthcare trust, the SensiumVitals® system inherits all the hospital procedures and data backup policies, ensuring data access and servers are secured.

In line with the principles of Good Clinical Practice guidelines, data will be securely archived for a minimum of 5 years.

Following the completion of quarantine, participants will be invited to share their experiences through using semi-structured interviews and questionnaires. Individual interviews allow for anonymity and truthful perceptions/attitudes. This method enables narratives to be elicited through guided but open questioning.

### Hypotheses

1. Continuous remote monitoring with wearable sensors of vital signs will identify deteriorating patients.
2. Healthcare provision in a hotel will be feasible and acceptable amongst healthcare staff and participants.

### Sequence of events

#### Patching patients and using data for academic research

1. Eligible participants from the airport will be transferred to the hotel by paramedics.
2. Upon arrival to the engineered hotel, an initial assessment of all participants will be undertaken. A study information sheet will be provided and informed consent taken for wearable sensors to be applied, allowing remote monitoring of vital signs, to ensure appropriate quarantine.
3. In a designated area within the building, a central monitoring hub will be set up to monitor the recorded parameters by the National Health Service (NHS) healthcare staff from a local Trust. The hub contains a site manager, porters, security staff, nurses, ambulance services, professional cleaners, and hotel staff.
4. If a participant deteriorates, an alert should be generated on a smartphone device/desktop computer; following this, an escalation protocol will be trialled to action the alert in an appropriate manner; hospitalisation may be necessary. Initially, nursing staff will gather more information through telephone review. Following this, a virtual GP can be contacted for further advice. Ambulance services and paramedics will be on site to facilitate hospitalisation should rapid deterioration occur. These healthcare professionals are independent and can provide oversight.
5. Participants will be invited for an interview and be asked to complete a survey on their experiences following quarantine completion.
6. Participant care records will be stored on an online Care Information Exchange system (compliant with General Data Protection Regulation) that is currently in place across the trust. Electronic health records and patient notes will be reviewed to determine clinically relevant events if transfer to hospital occurs (e.g. hospital length of stay, mortality, escalation to ITU).
7. Sensor data will be extracted from the central servers containing sensor data for appropriate statistical analyses for associated clinical efficacy of health outcomes in COVID-19.

#### Semi-structured interviews/questionnaires (mixed methods)

1. All participants alongside healthcare staff will be invited to take part in semi-structured interviews and be given questionnaires. These will take place by the primary lead researcher using prepared topic guides.
2. Written consent will be obtained from those who agree to participate.
3. An audio recording will be made of the interviews. Notes reflecting verbal responses may be taken. The interviews will be conducted at the engineered hotel.
4. The recordings will be transcribed, and the data will undergo thematic analyses.
5. Validated and unvalidated questionnaires will also be given enquiring about experiences (e.g. PHQ-9 [16], GAD-7 [17], satisfaction, anxiety related to the devices).

### Outcomes and progression criteria

#### Feasibility outcomes

1. Rate of participation
  Using the confidence interval approach [18], for a minimum sample size of 10 individuals, we estimate a rate of participation of 90% with a 95% confidence interval of ± 18%.
2. Generation of an alert following abnormal vital signs (e.g. raised temperature).
  We aim for a minimum of 5 vital alerts to be generated to demonstrate feasibility.
3. Missing data recorded from sensor limited to less than 20% attrition will be a criterion for progression.
4. Number of adverse events relating to the sensor system (e.g., skin reaction to sensor preventing trial continuation)

#### Exploratory clinical outcomes

1. Actions following alert generation (e.g. phone consultation, virtual general practitioner review, transfer to hospital).
2. Acceptability and usability of the SensiumVitals™ system by healthcare staff and participants (mixed methods analysis)

### Participant eligibility

The study will recruit flight arrivals returning to the UK in London. High-risk travellers are screened at the airport and tested for COVID-19, with transfer to hospital if appropriate; this cohort would be ineligible for the study.

Additionally, healthcare professionals who display symptoms of COVID-19 and are unable to isolate safely at home (e.g. lodging with vulnerable persons) would be eligible to take part in the study and invited to stay at the hotel.

#### Inclusion criteria

- Aged 18 years or over.
- Able to provide written consent.
- Travellers returning with milder symptoms suggestive of COVID-19 or returning from high-risk areas requiring quarantine, as recommended by the Public Health England.
- Healthcare professionals with milder symptoms suggestive of COVID-19 unable to isolate at home.

#### Exclusion criteria

- Any participants that withdraw their consent.
- A skin condition/reaction preventing wearing the wearable sensor (these can be communicated by the participant to the researcher or healthcare staff at the hotel).
- The presence of a permanent pacemaker or cardiac defibrillator.
- Any form of psychiatric disorder or a condition that, in the opinion of the investigator, may hinder communication with the research team.
- Inability to cooperate or communicate with the research team.

### Statistical considerations

#### Quantitative analysis

Descriptive statistics will be obtained about the baseline characteristics of participants. Continuous variables will be presented as mean ± standard deviation or medians and ranges, depending on distribution. Categorical variables will be reported as numbers and percentages. The total frequencies of alerts, proportion of actioned alerts and resultant actions will be described. Outcome measures (e.g. phone consultation, virtual GP review, transfer to hospital) will be reviewed from case notes should escalation occur and described in absolute frequencies. Questionnaire data will be presented using frequency distributions. Data will be analysed using SPSS, Stata and GraphPad.

#### Qualitative analyses

Semi-structured interviews will take place by the primary lead researcher using the prepared topic guides. An audio recording will be made of all interviews. The interviewer may also take field notes reflecting the verbal responses and reflections to be used to adapt the topic/study direction. The audio recordings will be transcribed. The data will be analysed appropriately using thematic analysis.

To ensure confidentiality and anonymity, each interview will be allocated a pseudonym, to be applied to the corresponding consent form, topic guide form/field notes and audio file. Their name will not be included in the audio recording; the pseudonym will be used.

Summaries of interview field notes will be typed into a word processor. All paper and soft copies of field notes, audio files and consent forms will be kept securely in a locker within a locked office and, if in digital format, on a password-protected computer and backed up regularly. Information will only be shared within the study team.

Audio recordings will be professionally transcribed verbatim, and a random selection of transcripts will be checked against recordings for accuracy. Interview transcripts will be analysed using Braun and Clarke’s thematic analysis by two independent researchers; disagreements will be resolved through discussion [19].

#### Power considerations

As a pilot study in an unknown viral pandemic, the progression of which is not well established; this study will appraise the feasibility for hotel remote sensing under these circumstances. Sufficient other data do not exist to allow for power calculations.

#### Patient and public involvement

Due to the nature of the pandemic and the current climate (national lockdown advised from the government) with COVID-19, patient and public involvement was not undertaken for this observational trial. All eligible participants will be approached to enter the study, and all recruited participants can provide email addresses should they wish for dissemination of results.

## Discussion

To the best of our knowledge, this is the first remote monitoring study focussing on healthcare delivery during a global pandemic at a remote site (i.e. hotel), acting as an extension to a healthcare trust. Apart from reducing infection risk to healthcare staff, continuous remote monitoring using a discrete wearable sensor has the potential to detect earlier clinical deterioration allowing for earlier intervention and provide further insight into the clinical course of COVID-19 in regards to vital signs.

Our pilot study design will test the viability of using a remote site for healthcare delivery during times of crisis. Continuous vital sign monitoring should provide insight to determine whether vital sign trends can detect clinical deterioration for COVID-19, requiring hospitalisation. Furthermore, questionnaires and semi-structured interviews of participants will provide insight into wider implementation of this technology and provide feedback for improvements. Similarly, semi-structured interviews of staff will provide a healthcare perspective, particularly thoughts on reducing potential infection risk through remote monitoring services.

Despite the strengths of our study, the design presents inherent limitations. Mainly, the lack of randomisation and control arm to compare remote-monitoring services to ‘standard care’. However, to maximise capacity at the hotel given the unknown of the pandemic, a pragmatic, observational design was favoured; randomisation was not deemed appropriate. Government restrictions are rapidly changing for air travel, which could significantly alter our sample size, increasing the risk of type II errors. The inclusion of healthcare professionals may bias the description of favourable experiences, owing to their familiarity of continuous monitoring of physiological parameters. In addition, implementing remote monitoring systems entails an initial financial cost with new logistical and legal considerations; given the early phase of work, the true extent of these issues remains unknown. If the trial is proven to be feasible, this work could power future randomised trials to explore for cause-effect relationships and describe cost-utility/effectiveness of remote monitoring solutions.

In conclusion, the results of our study would have potential to demonstrate the feasibility of remote monitoring during a pandemic and may provide insight into earlier recognition of clinical deterioration in individuals suspected with COVID-19.

## Data Availability

The datasets used and/or analysed during the current study are available from the corresponding author on reasonable request. Only the authors have access to the dataset.
